# A human memory circuit derived from brain lesions causing amnesia

**DOI:** 10.1038/s41467-019-11353-z

**Published:** 2019-08-02

**Authors:** Michael A. Ferguson, Chun Lim, Danielle Cooke, R. Ryan Darby, Ona Wu, Natalia S. Rost, Maurizio Corbetta, Jordan Grafman, Michael D. Fox

**Affiliations:** 1 Berenson-Allen Center for Noninvasive Brain Stimulation, Department of Neurology, Beth Israel Deaconess Medical Center, Boston, MA 02215 USA; 2 Harvard Medical School, Boston, MA 02115 USA; 3 Department of Neurology, Vanderbilt University Medical Center, Nashville, TN 37232 USA; 4 Athinoula A. Martinos Center for Biomedical Imaging, Massachusetts General Hospital, Charlestown, MA 02129 USA; 5 Stroke Research Center, Department of Neurology, Massachusetts General Hospital, Boston, MA 02114 USA; 6 Dipartimento di Neuroscienze, Università di Padova, Padova, 35122 Italy; 7 Departments of Neurology, Radiology, Neuroscience, and Bioengineering, Washington University, School of Medicine, St. Louis, 63110 USA; 8 Padova Neuroscience Center, Università di Padova, Padova, 35131 Italy; 9 Cognitive Neuroscience Laboratory, Think + Speak Lab, Shirley Ryan Ability Lab, 355 E Erie St., Chicago, 60611 USA; 10 Department of Physical Medicine and Rehabilitation, Feinberg School of Medicine, Northwestern University, Chicago, IL 60611 USA

**Keywords:** Hippocampus, Long-term memory, Spatial memory, Alzheimer's disease, Stroke

## Abstract

Human memory is thought to depend on a circuit of connected brain regions, but this hypothesis has not been directly tested. We derive a human memory circuit using 53 case reports of strokes causing amnesia and a map of the human connectome (*n* = 1000). This circuit is reproducible across discovery (*n* = 27) and replication (*n* = 26) cohorts and specific to lesions causing amnesia. Its hub is at the junction of the presubiculum and retrosplenial cortex. Connectivity with this single location defines a human brain circuit that incorporates > 95% of lesions causing amnesia. Lesion intersection with this circuit predicts memory scores in two independent datasets (N1 = 97, N2 = 176). This network aligns with neuroimaging correlates of episodic memory, abnormalities in Alzheimer’s disease, and brain stimulation sites reported to enhance memory in humans.

## Abstract (web summary)

Memory is hypothesised to depend on different brain regions that interact in a network. Here, the authors use case studies of stroke patients with amnesia from the literature to identify brain regions that are part of this network.

## Introduction

In 1937 James Papez described a human brain circuit based on gross pathology that included the hippocampus, anterior thalamus, mammillary bodies of the hypothalamus, posterior cingulate, and fornix^1^. Although initially described as an emotion circuit, it was later noted that lesions to this circuit disrupted episodic memory^2–4^. The best-known example is patient H.M., who suffered severe anterograde amnesia following bilateral medial temporal lobe resections^2,5^.

Subsequent work with laboratory animals^6–8^ and human neuroimaging^9–11^ further supported these findings, leading to general acceptance that memory (specifically episodic memory) localizes to the circuit of Papez. Neuroimaging studies have also identified a partially overlapping circuit, termed the default mode network, hypothesized to mediate episodic memory^10,12,13^.

The exact location of this circuit has taken on new importance with the increased prevalence of memory disorders such as Alzheimer’s disease^14,15^ and efforts to identify new therapies^16^. For example, brain stimulation aimed at improving human memory has been directed at multiple different brain targets, both inside and outside the traditional circuit of Papez^17–20^. These studies highlight important unanswered questions regarding the localization of human memory including whether memory localizes to a brain circuit, which regions should be included in this circuit, and whether some nodes of this circuit are more important than others^21^. In fact, the hypothesis that human brain lesions that disrupt memory localize to a single connected brain circuit has never been formally tested.

Recently, new tools have become available that allow us to better address these questions. By combining lesion locations with a wiring diagram of the human brain termed the human connectome, we can determine whether lesion locations causing similar symptoms fall within a single brain circuit and the hub of this circuit. This technique, termed lesion network mapping, has been successfully applied to hallucinations, delusions, movement disorders, coma, and even criminality^22–24^. Here, we apply this technique to lesions disrupting memory.

## Results

### Identifying amnesia-causing lesions

**Fig. 1:**
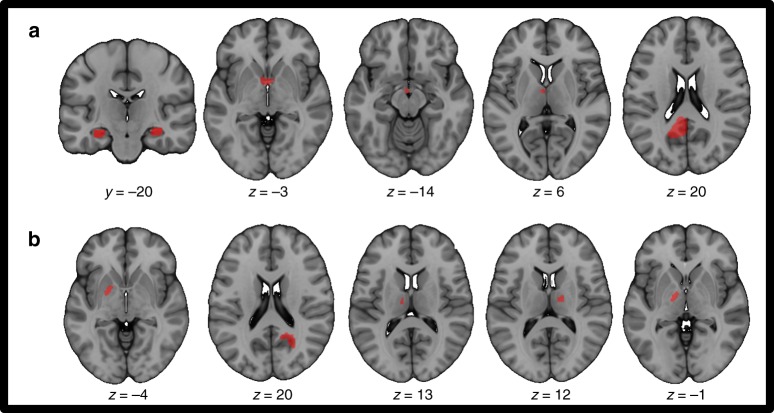
Ten examples of lesions causing amnesia (from total sample of 53). Lesions causing amnesia include lesions within the classic circuit of Papez (**a**) and lesions outside the classic circuit of Papez (**b**)

In our literature search for amnesia-causing lesions, we identified 53 lesion locations causing clinically-evident deficits in episodic memory (Supplementary Table 1). Lesion location was heterogeneous and included numerous different brain regions (Fig. 1). As expected, many lesions occurred at classic locations within the circuit of Papez (Fig. 1a); however, many lesions did not (Fig. 1b).

All 53 lesion cases were classified as “severe” amnesia (the memory deficit was clinically apparent even without formal neuropsychological testing), involved anterograde memory loss, and included documented impairment in verbal memory (Supplementary Table 1).

### Mapping amnesia-causing lesions to a common brain circuit

**Fig. 2:**
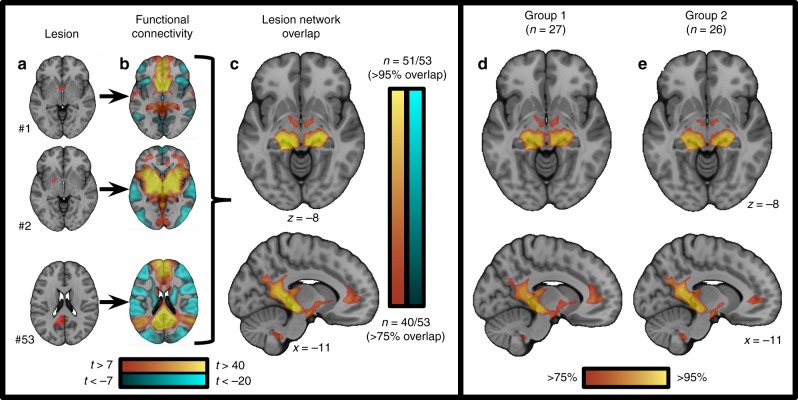
Lesion network mapping method and split half replication. Individual amnesia-causing lesions were mapped to a common brain template (**a**). Connectivity between each lesion location and the rest of the brain was computed using resting state functional connectivity from 1000 healthy control subjects (**b**). Positive correlations with the lesion location are shown in warm colors while negative correlations (anticorrelations) are shown in cool colors. Individual lesion network maps were thresholded, binarized, and overlapped to identify connections common to the lesion locations (**c**). Random splitting of our amnesia-causing lesion sample into two cohorts demonstrates high reproducibility of lesion network overlap (**d**, **e**). Additional iterations of random splitting were similar (Supplementary Figure 2)

We computed the network of brain regions functionally connected to each lesion location (Fig. 2). Despite heterogeneity in lesion location, lesions causing amnesia were part of a common brain circuit. Over 95% of amnesia-causing lesion locations were functionally connected to a single location in the hippocampus (Fig. 2c). The connectivity pattern of lesions causing amnesia was highly reproducible when split into two randomized subsamples (spatial correlation r = 0.98, Fig. 2d, e), and when restricting analysis to sub-cohorts with formal score reports, retrograde amnesia, and visual memory impairment (Supplementary Fig. 2).

**Fig. 3:**
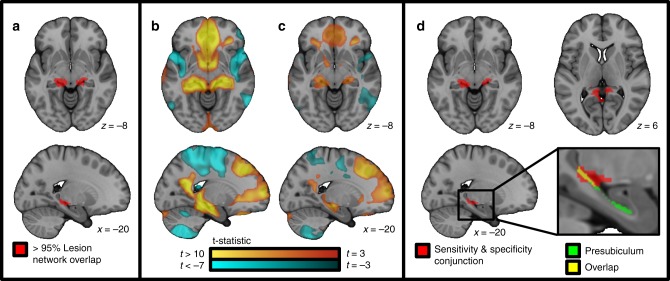
Sensitivity and specificity of lesion network mapping of amnesia. Using our full cohort of lesions causing amnesia (*n* = 53), >95% of lesion locations were functionally connected to the junction of the hippocampus and retrosplenial cortex (**a**). This connectivity was specific to lesions causing amnesia compared to a large cohort of control lesions causing non-specific symptoms (**b**) or lesions causing specific symptoms other than amnesia (**c**). (Specificity analyses compared unthresholded lesion network maps between amnesia and non-amnesia groups using a voxelwise two-sample *t*-test corrected for multiple comparisons with a conservative voxel-based Family Wise Error rate *p* < 0.05.) The conjunction of our sensitivity and specificity analyses identifies a focal region at the subiculum-retrosplenial continuum (**d**) that overlaps the posterior segment of the pre-subiculum

This connectivity profile was specific to lesions causing amnesia compared to generic lesions (Fig. 3b, two-sample *t*-test voxelwise FWE *p* < 0.05) or lesions causing other non-memory symptoms (Fig. 3c, two-sample *t*-test voxelwise FWE *p* < 0.05). A conjunction analysis identified a focal region in the subiculum-retrosplenial continuum whose connectivity was both sensitive (connected to >95% of amnesia lesions) and specific (two-sample *t*-test voxelwise FWE *p* < 0.05 in both specificity analyses) for lesions causing amnesia (Fig. 3d). Note that only 10 of our 53 amnesia lesions physically intersected this location, but 51 of our 53 lesions were functionally connected to this location. A small anticorrelated region in the intraparietal sulcus also survived this analysis (Supplementary Fig. 3).

**Fig. 4:**
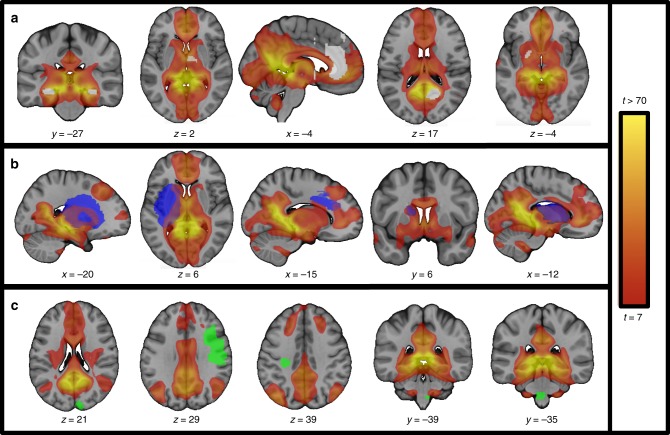
Validation of our human memory circuit in independent lesion cohorts. Functional connectivity with the subiculum-retrosplenial continuum (see Fig. 3d) defines a human memory network that by definition encompasses 51/53 lesion locations causing amnesia (**a**, lesion locations shown in white). Intersection between lesion location and this network was associated with memory scores in an independent lesion dataset (*n* = 97), including the five patients with the worst verbal memory scores (**b**, lesion locations shown in purple) and the five patients with the best verbal memory scores (**c**, lesion locations shown in green)

By definition, functional connectivity with our region in the subiculum-retrosplenial continuum defines a human brain circuit that encompasses >95% of lesion locations causing amnesia (Fig. 4a). This circuit includes classic nodes in the circuit of Papez and the default mode network, but also regions outside these networks (Fig. 4a, Table 1, Supplementary Fig. 4). For example, frontal and parietal cortical regions fall outside the classic circuit of Papez while cerebellar, thalamic, white matter, and occipital regions fall outside the classic default mode network.

**Table 1:** Locations of local maxima within our lesion-derived episodic memory network

Brodmann’s areas	Common names	MNI coordinates
27	Subiculum-retrosplenial junction	(−6, −41, 3), (8, −39, 3)
27	Subiculum	(−16, −31, −9), (16, −31, −9)
	Anterior medial thalamus	(−2, −7, 5), (2, −7, 5)
10	Ventral medial prefrontal cortex	(0, 51, −3)
19/39	Lateral parietal cortex	(−40, −75, 39), (48, −67, 35)
9	Superior frontal cortex	(−20, 33, 47), (24, 35, 45)
21	Lateral temporal cortex	(−62, −5, −13), (60, −1, −17)
17	Medial occipital cortex	(−2, −89, −3), (8, −89, 3)
	Corpus callosum	(−6, 5, 25), (8, 3, 27)
	Cerebellum, lobule IX	(−10, −45, −45), (12, −43, −45)

### Damage to circuit predicts memory in independent datasets

Overlap between lesion location and our memory circuit predicted continuous memory scores in an independent dataset of stroke lesions^25^ and in an independent dataset of lesions from penetrating head trauma^26^ (Fig. 4b, c). Immediately after stroke, intersection with our memory circuit was correlated with both verbal memory (Pearson correlation, r = −0.21, *p* < 0.04) and spatial memory scores (Pearson correlation, r = −0.36, *p* < 10^−3^). Following penetrating head trauma, intersection between lesion location and our memory circuit was correlated with remote memory scores for events that occurred close to the time of brain injury (Pearson correlation, r = −0.34, *p* < 10^−5^). All correlations remained significant after controlling for patient age, education, and lesion size (*p* < 0.05 for all analyses, Supplementary Table 2). Finally, we tested whether our memory circuit was a better predictor of lesion-induced memory deficits in these independent datasets than a priori maps of the default mode network. No matter which a priori map of the default mode network we used, which covariates we included, and which memory test we examined, intersection with our memory circuit was a significant independent predictor of lesion-induced memory deficits while intersection with the default network was not (*p* < 0.05 for all analyses, Supplementary Table 3).

Exploratory analyses restricted to brain lesions falling outside the classic circuit of Papez (e.g. in frontal and lateral parietal cortex) and outside the classic default mode network (e.g. in occipital cortex and brainstem), suggest that lesion locations intersecting peripheral nodes of our memory circuit are still associated with worse memory scores (spatial memory: Pearson correlation, r = −0.46, *p* < 5 × 10^−4^; verbal memory: Pearson correlation, r = −0.19, *p* < 0.17; Supplementary Fig. 5).

### Relevance beyond brain lesions

**Fig. 5:**
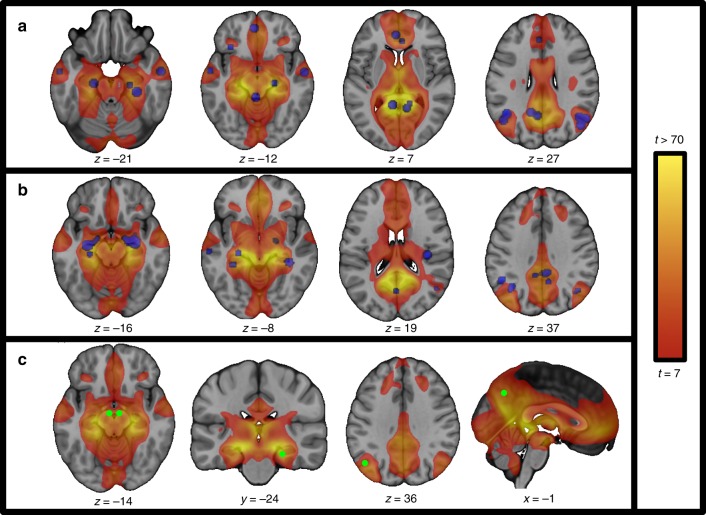
Memory circuit alignment with other imaging and stimulation modalities. Our human memory circuit derived from amnesia-causing brain lesions aligns well with data from other modalities. Functional connectivity with the subiculum-retrosplenial continuum (See Fig. 3D) defines a brain network that aligns well with neuroimaging correlates of episodic memory based on functional neuroimaging (**a**, purple spheres), neuroimaging abnormalities in Alzheimer’s disease (**b**, purple spheres), and locations where brain stimulation has been reported to enhance memory performance in humans (**c**, green spheres)

Our lesion-derived memory circuit aligned well with neuroimaging correlates of episodic memory in normal subjects (Fig. 5a) and neuroimaging abnormalities in Alzheimer’s disease (Fig. 5b). Overlap with neuroimaging correlates of episodic memory was significantly stronger than for neuroimaging correlates of working memory (t (39) = 3.5, *p* < 0.005. 95% CI [13.0, 47.7]) or language (t (39) = 3.4, *p* < 0.002, 95% CI [11.8, 46.6]; two-sample *t*-test, Bonferroni corrected). Overlap with neuroimaging abnormalities in Alzheimer’s disease was significantly greater than for neuroimaging abnormalities in frontotemporal dementia (t (45) = 4.6, *p* < 5 × 10 ^−5^, 95% CI [10.3, 26.7]) or primary progressive aphasia (t (39) = 2.9, *p* < 0.007, 95% CI [4.7, 30.0]; two-sample *t*-test, Bonferroni corrected).

We qualitatively explored the relationship between our lesion-derived memory circuit and brain stimulation sites reported to enhance memory in humans. Our search identified four stimulation sites meeting inclusion criteria^17–20^, all four of which clearly fell within our memory circuit (Fig. 5c).

## Discussion

There are three major findings in this paper. First, lesions causing amnesia are part of a single functionally connected brain circuit. Second, this memory circuit can be defined based on connectivity to a single brain region, the subiculum-retrosplenial continuum. Finally, this memory circuit may be relevant beyond brain lesions, as it aligns with neuroimaging correlates of episodic memory, neuroimaging abnormalities in Alzheimer’s disease, and brain stimulation studies.

The circuit of Papez^1^, and more recently the default mode network^10,12,13,27–31^, have been proposed as neuroanatomical substrates for human memory. The topography of our circuit aligns remarkably well with this prior work. In fact, the alignment is so good that refinements based on our paper could be considered incremental. However, the most novel aspect of our paper is not refining the topography of this circuit but linking this circuit to human memory in a causal way. Unlike functional neuroimaging, lesion studies allow for causal links between neuroanatomy and brain function^24,32^. The hypothesis that human brain lesions causing amnesia localize to a connected brain circuit has never been formally tested. Here, we derive a human episodic memory circuit empirically, without a priori assumptions about the relevant brain regions, based on brain lesions that causally disrupt human memory. The technique used to perform this analysis, termed lesion network mapping, is still relatively new but has been validated and proven useful across a wide range of neuropsychiatric symptoms^22–24^. However, the current paper goes beyond these prior studies as it is the first to validate lesion network mapping results by predicting deficits on a continuous basis in independent lesion datasets. By definition, our memory circuit encompasses the original lesion locations used to derive this circuit. However, this is no guarantee that intersection with our memory circuit would predict continuous memory scores in independent lesion datasets. The fact that our circuit predicted memory scores in two independent datasets differing in lesion etiology and method of memory assessment is an important validation of our results and of lesion network mapping more generally.

Despite strong similarity, the topography of our lesion-derived memory circuit is not identical to either the circuit of Papez or the default mode network as classically defined. Regions in frontal cortex, lateral parietal cortex, occipital cortex, cerebellum, basal ganglia, and white matter fall outside the traditional circuit of Papez, while regions in the anterior thalamus, basal ganglia, cerebellum, occipital cortex, white matter, and midbrain fall outside the standard default mode network. The role of many of these regions in human memory has remained controversial despite supporting evidence^13,25,33–38^. Our results suggest these regions may be important, as intersection with our lesion-based memory circuit, but not the default mode network, was an independent predictor of lesion-induced memory deficits. Similarly, peripheral nodes of our memory circuit outside the classic circuit of Papez still showed utility in predicting lesion-induced memory deficits (see Supplementary Fig. 5).

In addition to this memory circuit derived from positive functional connectivity, we observed that the intraparietal sulcus is anticorrelated with lesion locations that disrupt memory (Supplementary Fig. 3). It is possible that this region plays an antagonistic role in episodic memory, with increased activity actually impairing episodic memory. Such a hypothesis, while speculative, has some support from prior neuroimaging studies^13^, TMS studies^39^, and selective sparing of this region in AD^40^.

The hub of our human memory circuit is at the junction of the subiculum and the retrosplenial cortex, a region referred to as the subiculum-retrosplenial continuum^41,42^. Connectivity to this single brain location defines a human brain circuit that encompasses over 95% of our 53 lesions causing amnesia. It is not surprising that the hub of our human memory circuit resides in the hippocampus, but exactly which area of the hippocampus was unclear^43^. Historically, much more attention has been paid to CA1 than to the subiculum^44,45^, although recent evidence has provided increasing support for the importance of the subiculum-retrosplenial continuum^41,42,46,47^. Anatomically, the subiculum is the main outflow track of the hippocampus^6^ with widespread cortical connections^48-51^, which could explain its connectivity to the greatest number of brain lesions causing amnesia. However, connectivity to this region was also specific, as it was not connected to lesions that did not disrupt human memory. Interestingly, the subiculum-retrosplenial continuum seems to be particularly sensitive to the effects of diaschisis. For example, lesions to the anterior nucleus of the thalamus will produce long-term potentiation abnormalities in the subiculum-retrosplenial continuum^52^. Whether amnesia from brain lesions in our circuit comes from diachisis-like effects on the subiculum-retrosplenial continuum, or whether memory requires intact function of the entire circuit connected to this region is an important topic for future work.

Of our 53 amnesia lesions, only two cases were not connected to the subiculum-retrosplenial continuum (Coslett 1991 and Maeshima 2010 in Supplementary Table 1). One case had a severe language deficit, and the authors hypothesized that this may have been responsible for an apparent memory deficit (Coslett 1991 in Supplementary Table 1). The other case showed significant attention deficits, and the authors noted that symptoms differed from those typical of amnesia (Maeshima 2010 in Supplementary Table 1). Given the remarkable consistency across our other 51 lesion cases, we suspect other deficits may have led to apparent (but not actual) episodic memory impairment in these cases.

There are several important limitations. The 53 amnesia cases used to derive the circuit relied on existing case reports, subject to publication bias and often limited clinical assessment by the original authors. In fact, the two cases that fell outside our circuit were almost certainly not cases of lesion-induced amnesia on closer inspection. Similarly, our 53 lesion cases are not intended to be exhaustive. There are likely other lesion cases that met our criteria but that we failed to include. However, given the high reproducibility of our split cohort and sub-cohort analyses, it is unlikely that inclusion of further lesions or additional stratification based on clinical characteristics would alter our results. Similarly, clinical heterogeneity or errors in lesion tracing should bias us against the present findings of a common brain circuit. In our independent validation cohorts, we did not have pre-lesion memory testing to examine changes in memory directly associated with the lesion but were limited to a single assessment influenced by age, education, incipient disease, and undoubtedly many other factors. This limitation is most obvious for the penetrating head trauma dataset, in which memory was tested decades after the lesion, and the exact date of injury was unknown (making it unclear whether remote memory deficits from around the time of the lesion were anterograde or retrograde). Similarly, we were limited by the memory tests administered in these cohorts, limiting our ability to show specificity to episodic versus other forms of memory (e.g. semantic or implicit memory). Finally, we used resting state functional connectivity to define our circuit, consistent with prior lesion network mapping studies^22–24^, which is an indirect measure of neuronal activity with inherent limits in spatial resolution. For example, the subiculum-retrosplenial continuum lies in close anatomical proximity to the posterior cerebral artery which could introduce vascular artifact^51^. Finally, one could argue that functional connectivity data acquired during an episodic memory task (rather than during rest) would be more appropriate^52^. Future work using data from different episodic memory tasks to try and improve on our prediction of lesion-induced memory deficits should be considered. However, networks derived from resting state and task data are likely to be extremely similar^53–56^ and one must be careful to avoid confounds associated with task-based connectivity estimates^57,58^.

Our memory circuit was derived and validated using the lesion literature as focal brain lesions allow for causal inferences regarding localization of function^24,32,59^. However, our circuit is likely relevant for human memory in general, not just patients with brain lesions. First, our circuit aligns well with an extensive literature on neuroimaging correlates of episodic memory in normal subjects. In fact, several nodes of our circuit that go beyond the circuit of Papez (e.g. lateral parietal cortex, lateral temporal cortex) or the default mode network (e.g. midbrain, inferior frontal cortex) match these neuroimaging findings (see Fig. 5a). Second, our circuit aligns with pathology in other disorders of memory such as Alzheimer’s disease. While neuroimaging abnormalities in Alzheimer’s disease have not been consistent across different modalities^60,61^ these abnormalities map well to our memory circuit and to the default mode network^27,28^. Note that alignment of our memory circuit with neuroimaging correlates of episodic memory and Alzheimer’s pathology is not driven by the broad spatial extent of our memory circuit, as this alignment was specific compared to neuroimaging correlates of other functions or other diseases. Whether our memory circuit is a better predictor of Alzheimer’s pathology than the default mode network, and whether our circuit predicts memory deficits in Alzheimer’s as it does for brain lesions requires future work. Other disorders of memory including limbic encephalitis^62^ transient global amnesia^63^, and fentanyl overdose^64^ also appear to match our circuit well, although alignment with these pathologies was not formally tested.

Finally, our circuit aligns well with brain stimulation studies reported to enhance human memory^17–20^. Stimulation sites such as the fornix and medial temporal lobe are part of the classic circuit of Papez^17,18^, but lateral parietal cortex^19^ and precuneus^20^ are not, but still fall within our lesion-derived memory circuit. Future work is needed to determine whether our circuit can differentiate brain stimulation sites that do versus do not manipulate memory function. The current circuit topography could be useful as a guide for this future work, including providing a template for multifocal stimulation montages^65^.

## Methods

### Literature review and lesion tracing

The Medline database was searched through 2017 by combining the search terms “stroke,” or “cerebrovascular,” or “ischemia,” or “hemorrhage,” with the terms “amnesia,” or “memory”. The criteria not “subarachnoid,” not “dementia,” not “cardiac arrest,” not “transient global amnesia” were also added and search returns were limited to human studies. This search returned 4855 possible matches. These returns were limited to English language articles, and the titles of 1000 papers most related to the search criteria were reviewed, identifying the most relevant 500 papers. These abstracts were reviewed, identifying the most relevant English language papers. Abstract review looked for articles on human studies, primarily about a patient with memory loss, memory loss acquired by a lesion, and the etiology was not transient global amnesia, not Alzheimer’s disease-related, and not a brain tumor or other non-acquired lesions. From this set, we reviewed 250 full-text articles and included reports that fit the following criteria: (1) Case report format or individual case description; (2) Adult population; (3) Clinically relevant episodic memory deficits by bedside or neuropsychological tests attributed by the authors to an acute brain lesion; (4) Availability of a CT or MRI image depicting the lesion location(s) of sufficient quality that the lesion could be transcribed onto a standard brain template (Supplementary Fig. 1).

Fifty-three cases of amnesia were found with identifiable causative brain lesions (mean age 57.5 ± 13 years, range 27–72, 66% male). In three of 53 cases a second lesion was reported but was not thought to contribute to the acute memory deficit per the original authors (e.g. an old prior infarct) and was excluded. Brain lesions were mapped by hand onto a standard template brain from FSL (MNI152 asymmetric brain, 1 × 1 mm, http://fsl.fmrib.ox.ac.uk/fsldownloads/). Lesions from published figures were traced in the 2D plane(s) in which they were displayed, using neuroanatomical landmarks to accurately transfer the lesion location onto the template brain (Fig. 1).

### Lesion network mapping

The network of brain regions functionally connected to each lesion location was identified using a previously validated technique termed lesion network mapping^22–24^ (Fig. 2). Briefly, resting state functional connectivity between each lesion location and the rest of the brain was computed using a publicly available connectome dataset from 1000 healthy right-handed subjects (42.7% male subjects, ages 18–35 years, mean age 21.3 years)^28,66^. Resting state MRI data were processed in accordance with the strategy of Fox et al., 2005^27^, including global signal regression^67^. Both positive and negative correlations (anticorrelations) with the seed region of interest were included. Each of the 53 individual network maps was thresholded at a *t*-value of ±7 (corresponding to voxelwise FWE-corrected *p* < 10^−6^) consistent with prior work^23,68^. The resulting 53 network maps were binarized and overlapped to identify regions of shared connectivity and masked using a whole-brain template (MNI152 2009 asymmetric brain).

To test for reproducibility, the 53 amnesia-causing lesions were randomly sorted into two subgroups (Group A, *n* = 27; Group B, *n* = 26). Lesion network overlap maps were created separately for each subgroup, then compared using spatial correlation. This random splitting was performed multiple times to ensure reproducibility.

Next, we repeated our lesion network overlap analysis on lesion sub-cohorts including (1) cases with formal scores documenting amnesia severity (*n* = 30), (2) cases with documented retrograde memory impairment (*n* = 18), (3) cases with documented visual memory impairment (*n* = 20).

### Sensitivity and specificity testing

The lesion network overlap map for the full group (*n* = 53) was thresholded to identify regions connected to >95% of lesion locations causing amnesia (Fig. 3a). Specificity was assessed by comparing lesion network maps from lesion locations causing amnesia (*n* = 53) to network maps from lesion locations not specific to memory deficits (*n* = 490)^69^. A second specificity analysis was conducted using a separate set of control lesions (*n* = 63) selected and traced in the same way as the current amnesia lesions, but which were selected for causing an assortment of different neurological symptoms including aphasia, hallucinations, and pain^22^. Both specificity analyses compared unthresholded lesion network maps between groups using a voxelwise two-sample *t*-test in SPM 12, correcting for multiple comparisons with a conservative voxel-based Family Wise Error rate *p* < 0.05.

To identify an ROI both sensitive and specific to lesion locations causing amnesia, a conjunction between our sensitivity map (voxels connected to >95% of lesion locations causing amnesia) and voxels surviving both specificity tests was computed. The resultant ROI was overlapped with an existing hippocampal subfield atlas^70^.

### Defining a human episodic memory circuit

By definition, resting state functional connectivity with the above ROI defines a human brain circuit that encompasses lesion locations causing amnesia while avoiding control lesion locations. We derived this circuit using the above ROI as a seed region and the same resting state functional connectivity methods described earlier, including thresholding the map for significance at *t* > 7 (voxel-based FWE-corrected *p* < 10 ^−6^). Local maxima in this map were identified using the FSL clustering algorithm and a peak identification threshold of *t* > 26. We refer to this functional connectivity map as our lesion-based human episodic memory circuit.

### Validation using independent lesion datasets

To test the hypothesis that other lesions intersecting our lesion-based memory circuit would result in impaired memory, we used two independent lesion datasets in which formal memory testing was performed on each patient. Lesions in the first dataset (N1 = 97) were caused by ischemic stroke, with memory tests administered acutely following stroke^25^. We focused on acute factor scores for verbal and spatial memory based on prior work using this dataset^25^.

To ensure that results were not restricted to one lesion etiology (stroke), we repeated our analysis on a second independent dataset (N2 = 176) with lesions caused by penetrating head trauma during the Vietnam War. Memory tests were administered fifteen or more years after brain injury^26,71^. Memory for events around the time of brain injury was assessed using a previously published adaptation of the Marilyn Albert remote memory battery, restricted to events from the 1970s^71,72^.

We quantified the overlap between each lesion location and our memory circuit by adding the *t*-values of each voxel in our memory circuit that fell within each lesion mask. These values were then related to memory scores using Pearson correlation. Correlation analyses were repeated including age, education, and lesion size as covariates.

For visualization purposes, lesion locations from the five patients with the worst verbal memory factor scores and five patients with the best verbal memory factor scores (validation dataset 1, N1 = 97) were displayed overlaid on our human memory circuit.

To determine whether our memory circuit was a better predictor of lesion-induced memory deficits than the default mode network, we also computed lesion intersection with the default mode network. As there is no consensus on how best to define the default mode network, we defined it in three different ways based on previously published methods^27,28^ (Neurosynth meta-analysis term “default mode”). The default mode network seeds reported in Fox 2005^27^ and Yeo 2011^28^ were converted to default mode network maps using the same connectome that we used to generate our memory circuit and thresholded at *t* > 7. We compared the number of amnesia-causing lesions intersecting our lesion-derived memory circuit with the number of amnesia-causing lesions intersecting the three default mode network maps using a criteria of >50% of the lesion body intersecting the network of interest. Additionally, intersection between each lesion location and each default mode network map was quantified the same way we calculated intersection with our memory circuit. We then combined both measures of network intersection (memory circuit and default mode network) in a linear model to identify independent predictors of memory deficits. We repeated this analysis with age, education, and lesion size as covariates.

As an exploratory analysis, we tested whether lesions intersecting peripheral nodes of our memory circuit (i.e. regions outside the classic circuit of Papez) would also impair memory. We generated a subset of lesions from validation dataset 1 (*n* = 55 of 97) that fell outside the classic circuit of Papez by thresholding our lesion-based human memory circuit at *t* > 38 and selecting all lesions that failed to intersect any voxel. This threshold was chosen empirically by visual inspection to identify a set of lesions falling outside the classic circuit of Papez (Supplementary Fig. 5). The above Pearson correlation analyses were repeated on this lesion subset, focusing on acute verbal and spatial memory factor scores.

### Relationship to prior functional neuroimaging studies

MNI coordinates were extracted from previously published neuroimaging meta-analyses of episodic memory^73^ and Alzheimer’s disease^60^. As a control, coordinates were also extracted from meta-analyses of working memory^74^, language^75^, frontotemporal dementia^76^, and primary progressive aphasia^77^. For each coordinate, we identified the *t*-value in our lesion-based human memory circuit at that coordinate. The *t*-values for episodic memory^73^, were compared to those for working memory^74^, and language^75^ using two-tailed *t*-tests and Bonferroni correction (for two comparisons). A similar analysis was used to compare the *t*-values for Alzheimer disease^60^ to those for frontotemporal dementia^76^ and primary progressive aphasia^77^.

### Relationships to prior brain stimulation studies

A literature search was performed on PubMed using the search term combinations “brain stimulation memory enhancement”, “TMS Alzheimer memory”, and “deep brain stimulation Alzheimer memory”. Reports containing MNI coordinates of memory stimulation sites or a clear anatomical depiction of memory stimulation sites were selected^17–20^. Correspondence between previously reported stimulation sites and our lesion-based memory circuit was assessed qualitatively.

## Supplementary information

Supplementary Information

## Data Availability

The data that support the findings of this study are available from the corresponding author upon reasonable request.

## Code availability

The code that support the findings of this study are available from the corresponding author upon reasonable request.
