# Supplementary material for: A human memory circuit derived from brain lesions causing amnesia
Source: Nat Commun. 2019 Aug 2;10:3497. doi: 10.1038/s41467-019-11353-z (PMC6677746; doi:10.1038/s41467-019-11353-z)
Supplement: Supplementary file 1 — Supplementary Information [file 41467_2019_11353_MOESM1_ESM.pdf]

A human memory circuit derived from brain lesions causing amnesia

Ferguson et al.

SUPPLEMENTARY FIGURE 1

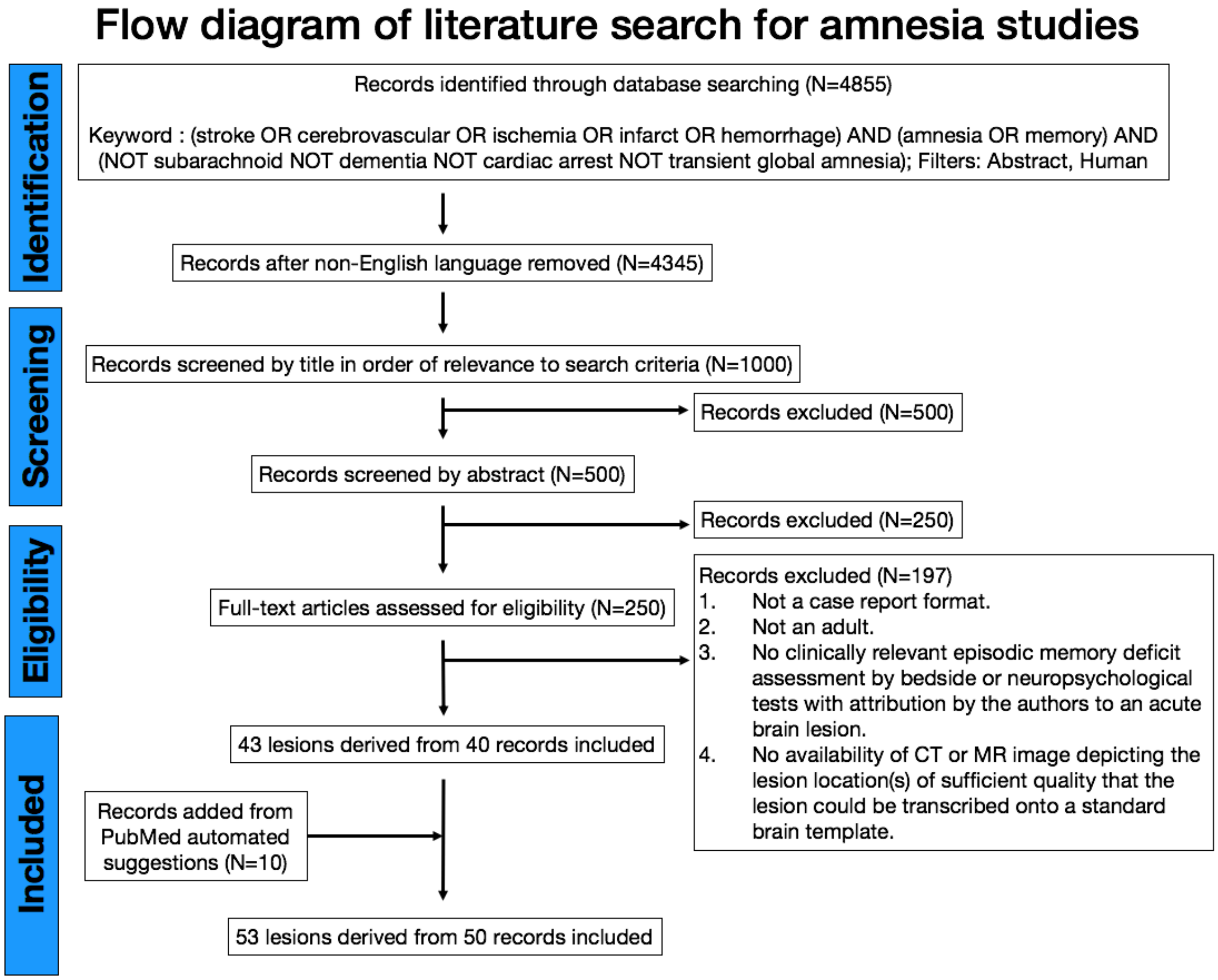

Supplementary Figure 1: Work flow for identifying cases of amnesia-causing lesions from the literature. 4,345 articles matched initial search criteria after filtering for English language articles. Of these, 1000 titles were reviewed in order of relevance to search criteria, 500 abstracts were reviewed and the 250 most likely to have imaging data for an amnesia case study were downloaded as candidate inclusion studies. Of these, 43 had suitable imaging for lesion network mapping. Ten additional cases were identified based on automated suggestions for related articles generated by PubMed or from references in the included studies. Our final set totaled 53 lesions, which is intended to be a representative but not exhaustive set of lesion locations causing amnesia.

SUPPLEMENTARY FIGURE 2

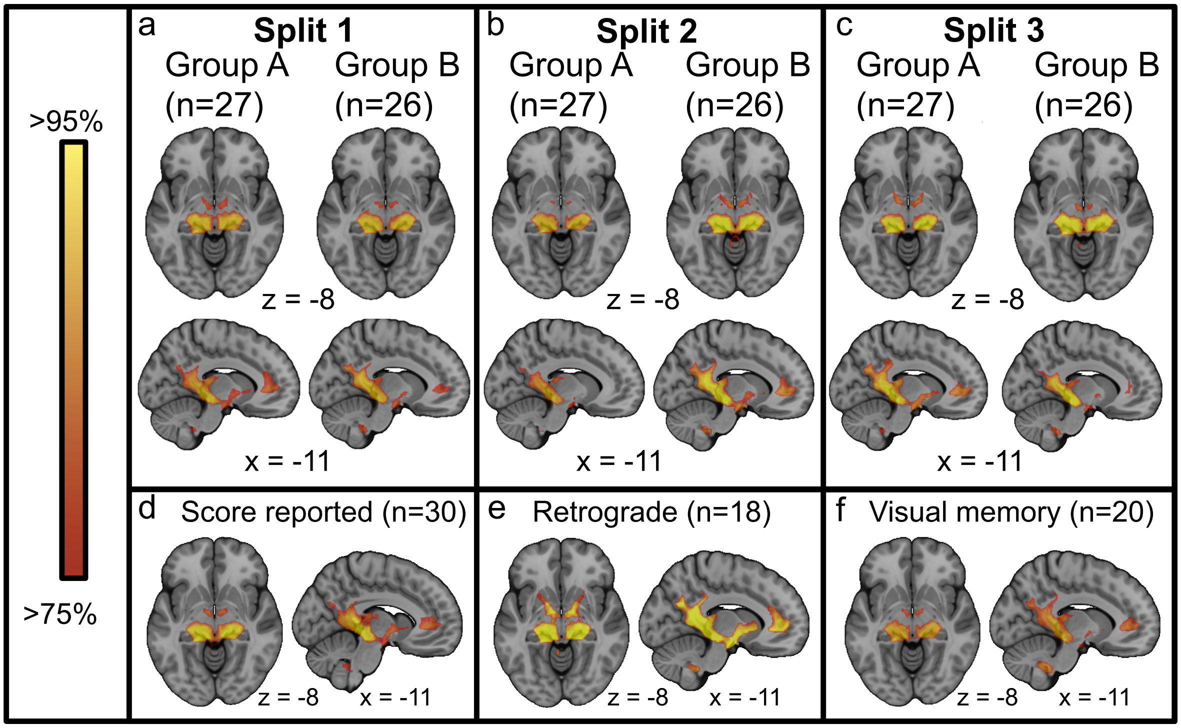

Supplementary Figure 2: Top row (a, b, c) Additional random splitting of our amnesia-causing lesion sample into sub-sampled cohorts demonstrates high reproducibility of lesion network overlap. **Bottom row (d, e, f)** We repeated our lesion network overlap analysis on lesion subcohorts including cases with formal scores documenting amnesia severity (n = 30; panel d), cases with documented retrograde memory impairment (n = 18; panel e), cases with documented visual memory impairment (n = 20; panel f). Lesion network overlap results were nearly identical across these sub-cohorts. For details of the scales and individual lesions comprising these sub-cohorts see Supplementary Table 1.

SUPPLEMENTARY FIGURE 3

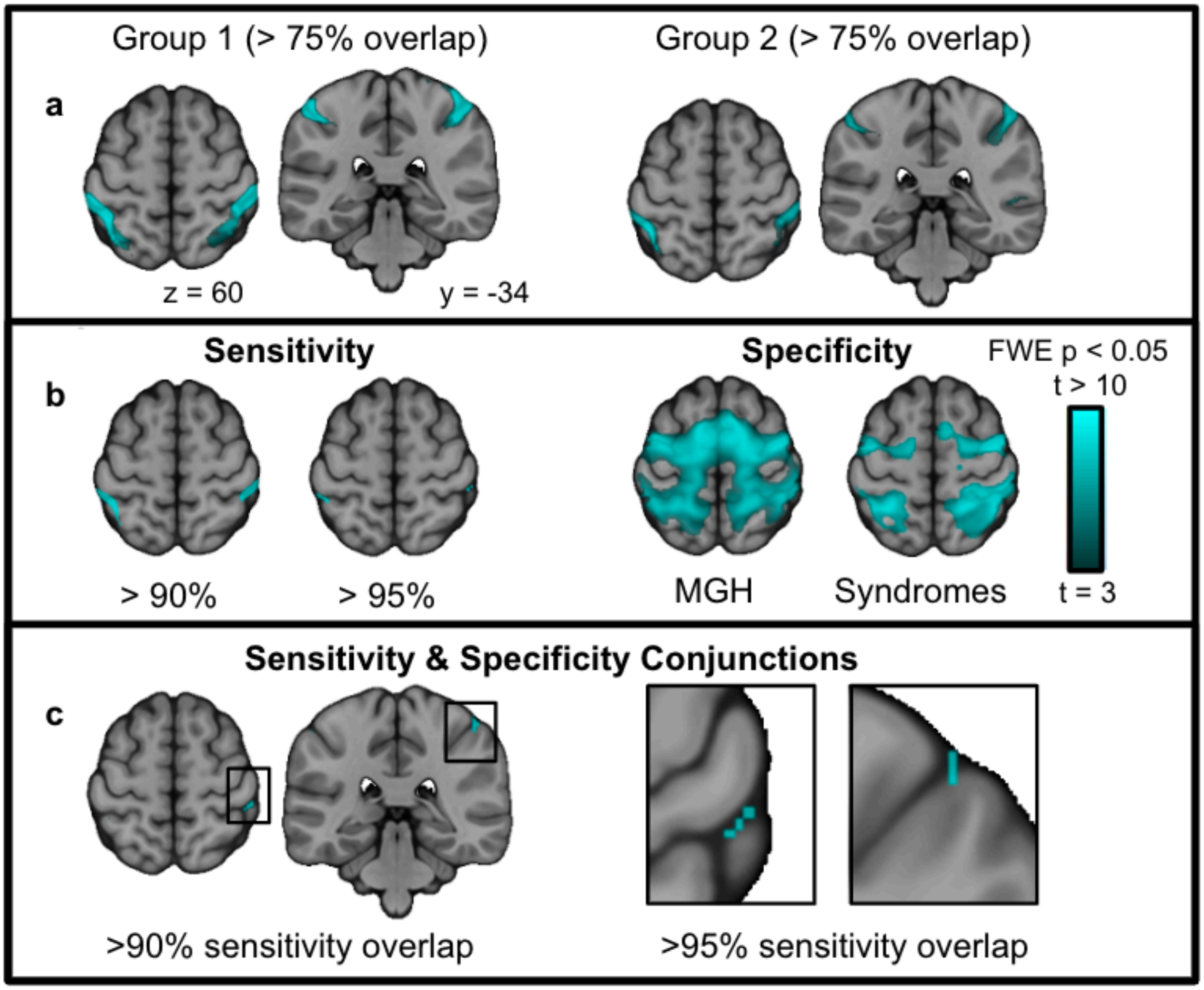

Supplementary Figure 3: Lesion network mapping of regions anticorrelated to lesions causing amnesia. **a)** Random splitting of our amnesia-causing lesion sample into two cohorts demonstrates high reproducibility of lesion network anticorrelation overlap. **b)** Across the whole sample of amnesia-causing lesions (n = 53) anticorrelation to memory network is highly sensitive (left) and demonstrates specificity in comparison with anticorrelations for non-specific lesions and with anticorrelations for lesions causing neurological syndromes (right). **c)** Conjunction of specificity and sensitivity tests are also depicted.

SUPPLEMENTARY FIGURE 4

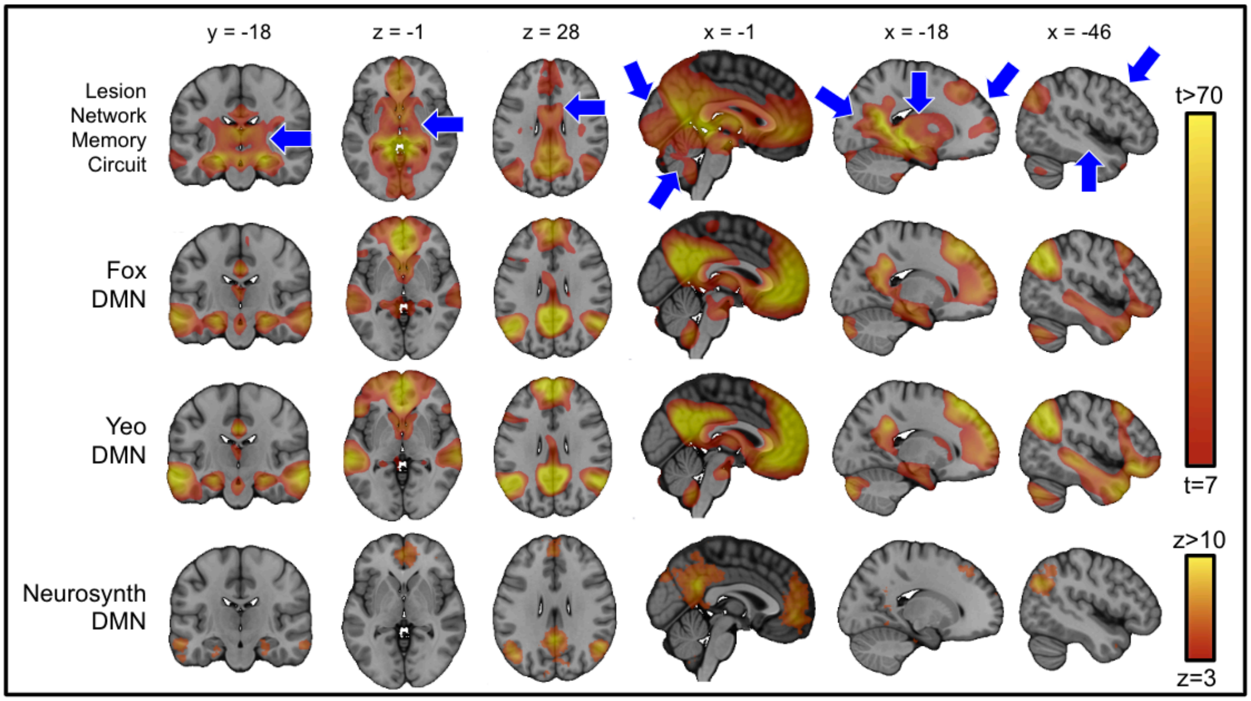

Supplementary Figure 4: Visual comparison of lesion network-derived memory circuit with the default mode network defined by three different strategies: Fox, 2005; Yeo, 2011; Neurosynth (term “default mode”). Blue arrows highlight areas of divergence between our lesion network-derived memory circuit and the default mode network.

SUPPLEMENTARY FIGURE 5

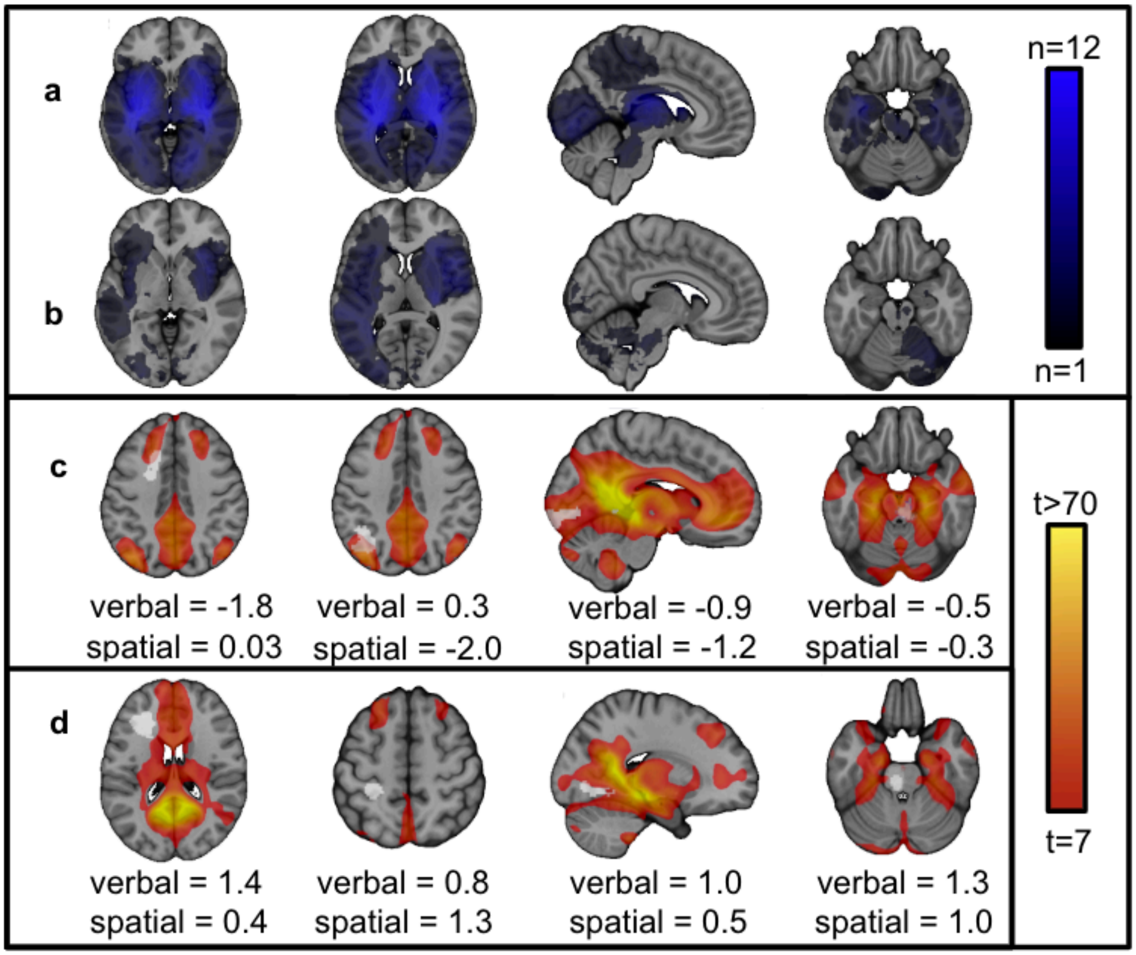

Supplementary Figure 5: Exploratory analysis of lesion locations intersecting peripheral nodes of our memory circuit. **a)** Lesions from validation dataset 1 (n = 97 ischemic strokes) that overlapped the core memory circuit (n = 42**).** MNI coordinates for displayed slices (L to R): z = -2; z = 8; x = 6; z = -21**.. b)** Lesions from validation dataset 1 that spared the core memory circuit (n = 55). MNI coordinates for displayed slices (L to R): z = -2; z = 8; x = 6; z = -21. **c & d)**: Four case / control examples of lesions that spared the core memory circuit and either overlapped **(c)** or spared **(b)** peripheral components of this circuit. MNI coordinates for displayed slices in row c (L to R): z = 41; z = 40; x = 8; z = -21. MNI coordinates for displayed slices in row d (L to R): z = 19; z = 56; x = 19; z = -28. Acute factor scores for verbal and spatial memory are shown below each lesion (negative values indicate worse memory). All lesions are shown overlaid on our memory circuit defined by functional connectivity with the subiculum-retrosplenial junction. All lesions are unilateral except the two brainstem lesions and the occipital lesion in row **a**.

SUPPLEMENTARY TABLE 1

# of cases	References for amnesia case studies meeting inclusion criteria	Score report	Retro-grade	Visual mem
1	Abe, K., Inokawa, M., Kashiwagi, A. and Yanagihara, T., 1998. Amnesia after a discrete basal forebrain lesion. Journal of Neurology, Neurosurgery & Psychiatry, 65(1), pp.126-130.	Y	Y	Y
1	Adamovich, B.L., Gualberto, G., Roberts, T., Haut, M.W. and Gutmann, L., 2009. Teaching NeuroImages: amnesia due to fornix infarction. *Neurology*, *73*(17), pp.e86-e86.		Y	
1	Amuluru, K., Filippi, C.G. and Lignelli, A., 2015. Acute amnesia due to isolated mammillary body infarct. *Journal of Stroke and Cerebrovascular Diseases*, *24*(10), pp.e303-e305.			
1	Bereznyakova, O., Milot, G., Brisson, M. and Laforce, R., 2014. Clinical Reasoning: A puzzling case of amnesia. Neurology, 83(6), pp.e73-e76.	Y	Y	
1	Bird, C.M., Shallice, T. and Cipolotti, L., 2007. Fractionation of memory in medial temporal lobe amnesia. Neuropsychologia, 45(6), pp.1160-1171.	Y		Y
1	Chen, J.Y., Zayas, V. and Gold, R., 2008. Anterograde amnesia and fornix infarction. Medicine and health, Rhode Island, 91(8), pp.258-258.		Y	
1	Clarke, S., Assal, G., Bogousslavsky, J., Regli, F., Townsend, D.W., Leenders, K.L. and Blecic, S., 1994. Pure amnesia after unilateral left polar thalamic infarct: topographic and sequential neuropsychological and metabolic (PET) correlations. Journal of Neurology, Neurosurgery & Psychiatry, 57(1), pp.27-34.	Y	Y	Y
1	Coslett, H.B., Bowers, D., Verfaellie, M. and Heilman, K.M., 1991. Frontal verbal amnesia: Phonological amnesia. Archives of Neurology, 48(9), pp.949-955.	Y		Y
3	de Oliveira Lanna, M.E., Alves, C.E.O., Sudo, F.K., Alves, G., Valente, L., Moreira, D.M., Cavalcanti, J.L.S. and Engelhardt, E., 2012. Cognitive disconnective syndrome by single strategic strokes in vascular dementia. *Journal of the neurological sciences*, *322*(1-2), pp.176-183.	Y		
1	Goldenberg, G., Wimmer, A. and Maly, J., 1983. Amnesic syndrome with a unilateral thalamic lesion: A case report. *Journal of Neurology*, *229*(2), pp.79-86.	Y		Y
1	Grewal, R.P., 2003. Severe amnesia following a unilateral temporal lobe stroke. Journal of clinical neuroscience, 10(1), pp.102-104.	Y	Y	
1	Hankey, G.J. and Stewart-Wynne, E.G., 1988. Amnesia following thalamic hemorrhage. Another stroke syndrome. Stroke, 19(6), pp.776-778.	Y		Y
1	Kim, S.H., Park, K.H., Sung, Y.H., Lee, Y.B., Park, H.M. and Shin, D.J., 2008. Dementia mimicking a sudden cognitive and behavioral change induced by left globus pallidus infarction: review of two cases.	Y		Y

	Journal of the neurological sciences, 272(1-2), pp.178-182.			
1	Kim, M.H., Hong, S.B. and Roh, J.K., 1994. Amnesia syndrome following left anterior thalamic infarction; With intrahemispheric and crossed cerebro-cerebellar. *Journal of Korean medical science*, *9*(5), pp.427-431.		Y	Y
1	Kooistra, C.A. and Heilman, K.M., 1988. Memory loss from a subcortical white matter infarct. Journal of Neurology, Neurosurgery & Psychiatry, 51(6), pp.866-869.	Y		
1	Korematsu, K., Hori, T., Morioka, M. and Kuratsu, J.I., 2010. Memory impairment due to a small unilateral infarction of the fornix. *Clinical neurology and neurosurgery*, *112*(2), pp.164-166.	Y	Y	
1	Kostić, S., Pasovski, V., Krsmanović, Ž., Bošković, Ž., Kostić, D., Jovanovski, A. and Jović-Stošić, J., 2015. Bilateral hippocampal infarction and amnesia: A case report. *Vojnosanitetski pregled*, *72*(6), pp.545-551.	Y		Y
1	Koutsouraki, E., Xiromerisiou, G., Costa, V. and Baloyannis, S., 2009. Acute bilateral thalamic infarction as a cause of acute dementia and hypophonia after occlusion of the artery of Percheron. Journal of the neurological sciences, 283(1-2), pp.175-177.			
1	Laplane, D., Degos, J.D., Baulac, M. and Gray, F., 1981. Bilateral infarction of the anterior cingulate gyri and of the fornices: report of a case. *Journal of the Neurological Sciences*, *51*(2), pp.289-300.		Y	
1	Lobaz, J. and White, J.R., 2012. Images in...:'Where am I?'–An unusual stroke presentation. BMJ case reports, 2012.			
1	Maeshima, S., Osawa, A., Yamane, F., Yoshihara, T., Kanazawa, R. and Ishihara, S., 2014. Retrosplenial amnesia without topographic disorientation caused by a lesion in the nondominant hemisphere. Journal of Stroke and Cerebrovascular Diseases, 23(3), pp.441-445.			
1	Maeshima, S., Ozaki, F., Masuo, O., Yamaga, H., Okita, R. and Moriwaki, H., 2001. Memory impairment and spatial disorientation following a left retrosplenial lesion. *Journal of clinical neuroscience*, *8*(5), pp.450-451.	Y		Y
1	Maeshima, S., Osawa, A. and Kunishio, K., 2010. Cognitive dysfunction in a patient with brainstem hemorrhage. Neurological sciences, 31(4), pp.495-499.	Y		Y
1	Maeshima, S., Ueyoshi, A., Tsuura, M., Takemoto, H., Itakura, T., Yoshida, M. and Matsumoto, T., 2002. Transient aphasia and persistent amnesia after surgery for internal carotid artery–posterior communicating artery aneurysm. *Journal of clinical neuroscience*, *9*(6), pp.710-713.	Y		Y
1	Male, S. and Zand, R., 2017. Isolated mammillary body infarct causing global amnesia: a case report. Journal of Stroke and Cerebrovascular Diseases, 26(3), pp.e50-e52.		Y	

1	Masuo, O., Maeshima, S., Kubo, K., Terada, T., Nakai, K., Itakura, T. and Komai, N., 1999. A case of amnestic syndrome caused by a subcortical haematoma in the right occipital lobe. Brain injury, 13(3), pp.213-216.	Y		Y
1	Meissner, I., Sapir, S., Kokmen, E. and Stein, S.D., 1987. The paramedian diencephalic syndrome: a dynamic phenomenon. *Stroke*, *18*(2), pp.380-385.	Y		
1	Mendizabal, J.E., Greiner, F., Hamilton, W.J. and Rothrock, J.F., 1997. Migrainous stroke causing thalamic infarction and amnesia during treatment with propranolol. Headache: The Journal of Head and Face Pain, 37(9), pp.594-596.			
1	Miranda, M.O., Suarez, E.M.A., Frutos, R., Vera, J.E., Ferrer, F.P. and Prado, J.A.L., 2015. Amnestic syndrome of the subcallosal artery with additional penetrating vessel involvement. Journal of the neurological sciences, 359(1), pp.438-439.			
1	Moudgil, S.S., Azzouz, M., Al-Azzaz, A., Haut, M. and Gutmann, L., 2000. Amnesia due to fornix infarction. Stroke, 31(6), pp.1418-1419.	Y		Y
1	Moussouttas, M., Giacino, J. and Papamitsakis, N., 2005. Amnestic syndrome of the subcallosal artery: a novel infarct syndrome. Cerebrovascular Diseases, 19(6), pp.410-414.	Y		Y
2	Ott, B.R. and Saver, J.L., 1993. Unilateral amnesic stroke. Six new cases and a review of the literature. *Stroke*, *24*(7), pp.1033-1042.			
1	Park, S.A., Hahn, J.H., Kim, J.I., Na, D.L. and Huh, K., 2000. Memory deficits after bilateral anterior fornix infarction. *Neurology*, *54*(6), pp.1379-1382.	Y		Y
1	Parkin, A.J., Rees, J.E., Hunkin, N.M. and Rose, P.E., 1994. Impairment of memory following discrete thalamic infarction. *Neuropsychologia*, *32*(1), pp.39-51.	Y		
1	Renou, P., Ducreux, D., Batouche, F. and Denier, C., 2008. Pure and acute Korsakoff syndrome due to a bilateral anterior fornix infarction: a diffusion tensor tractography study. Archives of neurology, 65(9), pp.1252-1253.		Y	
1	Rizek, P., Pasternak, S., Leung, A. and Jenkins, M.E., 2013. Acute-onset anterograde amnesia caused by isolated bilateral fornix infarction. Canadian Journal of Neurological Sciences, 40(5), pp.738-739.			
1	Ryan, D., Murphy, S.M. and Hennessey, M.J., 2010. Other full case: Bilateral posterior cerebral artery infarction. BMJ case reports, 2010.			
1	Saito, Y., Matsumura, K. and Shimizu, T., 2006. Anterograde amnesia associated with infarction of the anterior fornix and genu of the corpus callosum. *Journal of Stroke and Cerebrovascular Diseases*, *15*(4), pp.176-177.	Y		Y
1	Szabo, K., Förster, A., Jäger, T., Kern, R., Griebe, M., Hennerici, M.G. and Gass, A., 2009. Hippocampal lesion patterns in acute posterior cerebral artery stroke: clinical and MRI findings. Stroke, 40(6), pp.2042-2045.			

1	Stenset, V., Grambaite, R., Reinvang, I., Hessen, E., Cappelen, T., Bjørnerud, A., Gjerstad, L. and Fladby, T., 2007. Diaschisis after thalamic stroke: a comparison of metabolic and structural changes in a patient with amnesic syndrome. *Acta Neurologica Scandinavica*, *115*, pp.68-71.			
1	Suzuki, K., Takei, N., Toyoda, T., Iwata, Y., Hoshino, R., Minabe, Y. and Mori, N., 2003. Auditory hallucinations and cognitive impairment in a patient with a lesion restricted to the hippocampus. Schizophrenia research, 64(1), pp.87-89.			
1	Swanson, R.A. and Schmidley, J.W., 1985. Amnestic syndrome and vertical gaze palsy: early detection of bilateral thalamic infarction by CT and NMR. *Stroke*, *16*(5), pp.823-827.		Y	
1	Takamatsu, K., Yamamoto, M., Yamano, T. and Ohno, F., 1990. A case of amnestic syndrome due to right thalamic infarction. *Japanese journal of medicine*, *29*(3), pp.301-304.	Y	Y	Y
1	Turnbull, O.H. and Evans, C.E., 2006. Preserved complex emotion-based learning in amnesia. Neuropsychologia, 44(2), pp.300-306.	Y		
1	Valenstein, E., Bowers, D., Verfaellie, M., Heilman, K.M., Day, A. and Watson, R.T., 1987. Retrosplenial amnesia. *Brain*, *110*(6), pp.1631-1646.	Y	Y	Y
1	von Cramon, D.Y. and Schuri, U., 1992. The septo-hippocampal pathways and their relevance to human memory: a case report. *Cortex*, *28*(3), pp.411-422.			
1	Vucic, S., Lye, T. and Mackenzie, R.A., 2003. Neuropsychological manifestations in a case of bilateral thalamic infarction. *Journal of clinical neuroscience*, *10*(2), pp.238-242.	Y	Y	
1	Warren, J.D. and Thompson, P.D., 2000. Diencephalic amnesia and apraxia after left thalamic infarction. *Journal of Neurology, Neurosurgery & Psychiatry*, *68*(2), pp.248-249.		Y	
1	Yasuda, Y., Watanabe, T., Tanaka, H., Tadashi, I. and Akiguchi, I., 1997. Amnesia following infarction in the right retrosplenial region. *Clinical neurology and neurosurgery*, *99*(2), pp.102-105.	Y	Y	Y
1	Yoneoka, Y., Takeda, N., Inoue, A., Ibuchi, Y., Kumagai, T., Sugai, T., Takeda, K.I. and Ueda, K., 2004. Acute Korsakoff syndrome following mammillothalamic tract infarction. *American journal of neuroradiology*, *25*(6), pp.964-968.		Y	

Supplementary Table 1: References for the 53 case studies meeting inclusion criteria for our analysis, taken from 50 unique journal articles. All 53 lesion cases were classified as “severe” amnesia (the memory deficit was clinically apparent even without formal neuropsychological testing), involved anterograde memory loss, and included documented impairment in verbal memory. 30/53 cases reported a formal measure of amnesia severity, but the metrics varied. The most common metric was the Weschler Memory Scale general score (13 cases) followed by Cambridge Cognitive Examination memory score (five cases). Only nine cases provided both a Wechsler Memory Scale

general score and IQ score to allow for the calculation of a WMS discrepancy score. 19/53 cases reported whether there was some element of retrograde amnesia: 18/53 reported impairment while one reported that retrograde memory was intact. Only one case reported a score for retrograde amnesia via neuropsychological testing. 20/53 cases reported whether visual memory was impaired, all 20 of which reported impairment. Seven cases reported formal scores for visual memory impairment using the Benton Visual Memory Task. Finally, other characteristics of amnesia were rarely reported such as impairment in sematic memory (four cases), autobiographical memory (two cases), or temporal order memory (one case).

SUPPLEMENTARY TABLE 2

	r (full corr)	p (full corr)	p (partial corr)
**DATASET 1 (N1 = 97)**			
Verbal Memory Factor	-0.21	0.039	0.002
Spatial Memory Factor	-0.36	$< 4 \times 10^{-4}$	0.025
**DATASET 2 (N2 = 176)**			
Remote Memory	-0.34	6×10^{-6}	0.006

Supplementary Table 2: Overlap between lesion location and our memory circuit was correlated with memory scores in two independent lesion datasets (Corbetta et al., 2015) (Raymont et al., 2011). Partial correlation controlled for age, education, and lesion size as covariates. All p-values are reported as two-tailed measures of significance.

SUPPLEMENTARY TABLE 3

	Verbal memory	Spatial memory	Remote memory
**Test 1**	**N1 = 97**	**N1 = 97**	**N2 = 176**
Memory circuit	p = 0.004	p = 0.008	p = 0.03
DMN, Fox 2005	p = 0.07	p = 0.05	p = 0.49
**Test 2**			
Memory circuit	p = 0.01	p = 0.03	p = 0.03
DMN, Yeo 2011	p = 0.08	p = 0.05	p = 0.16
**Test 3**			
Memory circuit	p = 0.01	p = 0.03	p = 0.01
DMN, Neurosynth	p = 0.13	p = 0.10	p = 0.79

Supplementary Table 3: Comparison of memory circuit damage scores with default mode network damage scores demonstrates that memory circuit damage but not default mode network damage is an independent predictor of clinical memory scores. Memory circuit damage was tested against damage to the default mode network using three different strategies to different default mode network: Fox, 2005 (Test 1); Yeo, 2011 (Test 2); Neurosynth “default mode” (Test 3). Age, education, and lesion size are included as covariates in clinical memory score predictions. All p-values are reported as two-tailed measures of significance.
